# District nurses’ experiences of giving initial health care assessment to young adults applying for mental illness in primary care: a qualitative interview study

**DOI:** 10.1017/S146342362300018X

**Published:** 2023-04-17

**Authors:** Charlotte Östangård Olofsson, Ulrica Lovén Wickman

**Affiliations:** 1 Advanced nurse Primary care, Department of Health and Caring Sciences, Linnaeus University and Department of Primary care, Region Kalmar, Kalmar, Sweden; 2 Advanced nurse Primary care, PhD, Associate Professor, Department of Health and Caring Sciences, Linnaeus University, Kalmar, Sweden

**Keywords:** district nurse, experience, mental illness, primary care, young adults

## Abstract

**Background:**

Mental illness among young adults is increasing both nationally and internationally. Primary care’s mission is to be the hub of health care and to offer high-quality care regardless of age, patient group, or disease. The role of district nurse varies in terms of definition and scope of practice but has a central role through their health promotion mission and by being the first person these young adults meet in primary care.

**Aim:**

The aim of this study was to explore district nurses’ experiences of meeting young adults with mental illness in primary care.

**Method:**

The study was conducted with a qualitative inductive approach. Data were collected through semi-structured interviews with nine district nurses in primary care in Sweden. The data were analysed through qualitative content analysis.

**Results:**

Three categories emerged in the results – *The difficult meeting*, *The district nurse’s ability to promote health*, and *A sense of inadequacy*. The results show that district nurses can experience feelings of stress and frustration when time, resources, and knowledge are lacking. Continuous training in mental illness was desired by all district nurses. Listening, confirming, and daring to ask were highlighted as particularly important in the meeting with young adults. Cooperation between different professions and colleagues was highlighted as a prerequisite for the provision of good quality care.

**Conclusion:**

To meet the increasing number of young adults with mental health problems seeking care, district nurses in primary care need continuous training. By working in a person-centred and health-promoting manner, the district nurse’s competence can be utilized. The district nurses perceive they could manage the important role being a resource and take a greater responsibility to guide young adults on the right path.

## Introduction

Mental illness is on the rise worldwide, and suicide is now the second most common cause of death among young adults between the ages of 15 and 29 years globally. Mental illness is considered to have a significant effect on all aspects of life such as school, work, relationships, and the opportunity to be a part of society (WHO, 2021a).

## Background

### The concept of mental illness

Mental health includes the concepts of mental well-being and mental illness. Mental illness is a collective term to which both mental disorders and psychiatric conditions belong. Mental illness can be used as a term covering a broad range of mental health problems. However, those who suffer from mental illness often have difficulty functioning well in everyday life, and their illness can affect social relationships and the ability to work or study. The problems are of varying length and severity, and examples of mental illness can be anxiety, depression, schizophrenia, autism spectrum disorder, obsessive-compulsive disorder, eating disorders, personality disorders, attention-deficit hyperactivity disorder, alcohol disorder, difficulty concentrating, difficulty sleeping, self-hatred, and suicidal thoughts (Liu *et al.,* 2017; World Health Organisation, 2021b). In this study, mental illness is used in this wide perspective to include all mental illness that make it hard for young adults to function well in daily life.

### The mission of primary care

According to the National Board of Health and Welfare’s national guidelines for care for depression and anxiety syndrome (National Board of Health and Welfare, 2021), most young adults who suffer from depression or anxiety seek primary care. Over 70% of these are cared for in primary care and around 20% are referred to specialized psychiatry. Haddad *et al.,* 2007 have expressed the increase of mental illness and the district nurses increasing frequency of encounters with young adults with mental illness in primary care. Providing mental health services to young adults is important. However, health care professionals are shown to lack training and competence to handle mental health issues in general and specifically suicidal problems among young adults (Obando-Medina *et al.*, 2014). Primary care should be the basis of the health care system with a special mission for health promotion and for coordinating a coherent health care system. Broad competence in both physical and mental health as well as current routines and knowledge support among the health care professionals who first meet the patient is crucial (Ministry of Social Affairs, 2021).

### The role of the district nurse

The role of district nurse varies in terms of definition and scope of practice. To make mental health care more accessible, it is important to have a well-qualified workforce within primary care settings. Nurses in primary care embrace the autonomy of the role and the ability to engage with patients by providing clinical interventions that can assess and intervene with persons experiencing mental illness (Olasoji *et al.,* 2020). District nurses have contact with a wide range of mental health problems, and the role in caring is substantial. The district nurse should independently promote health by leading the work with lifestyle changes regarding dietary advice, physical activity, alcohol abuse, and tobacco cessation, as well as working with issues related to stress, sleep, and mental illness (Swedish Nurses’ Association, 2019). By taking a person-centred approach, the meeting with patients and relatives can take place with sensitivity, presence, and confirmation, which promotes participation and the ability to provide self-care. Good knowledge of conversational methodology is important to be able to supervise and provide advice and support. The meeting in this study refer to the district nurses experience of the encounter with the young adults by phone, digitally or face to face in Swedish primary care context at a health care centre.

Mental illness is a growing health problem, especially among young people between the ages of 16 and 29 years. Every third visit to primary care is made by people who have some type of mental illness. The district nurse in primary care is often the first person, young adults who apply for mental illness meet, either by telephone, digitally, or in a physical meeting. Primary care are the basis of the health and medical care system, which places higher demands on the district nurse. Previous research in the field indicates that lack of time, resources, and knowledge make it difficult to meet young adults. The number of young adults seeking primary care due to mental illness is increasing, and few studies have been conducted with a focus on district nurses’ experiences of these meetings.

## Aim

The aim was to explore district nurses’ experiences of meeting young adults with mental illness in primary care.

## Method

### Design

A descriptive qualitative study design with individual interviews was chosen that aimed to describe, explain, and interpret the district nurses’ experiences. The design is appropriate to describe variations in relation to similarities and differences (Polit and Beck, 2021). The consolidated criteria for reporting qualitative research (COREQ) were used for comprehensive reporting (Tong *et al.*, 2007).

### Setting

Health care centres from the southeastern and northern part of Sweden located in either rural (n = 6) or urban (n = 3) municipalities were asked to participate. Of these, five managers agreed to participate, and these health care centres were regionally controlled. The population varying between 6.000 and 64.000 inhabitants.

### Informants

The strategic selection was used to recruit informants to the study to ensure that district nurses with broad experience in the subject were included. Inclusion criteria were being a registered nurse with specialist training as a district nurse and with employment in primary care. District nurses employed within the unit received information letters, and those who agreed to participate were contacted via email or telephone to book an appointment for an interview.

In total, nine district nurses, all of whom were women between 31 and 60 years (median 44 years) with a work experience as a district nurse between 1 and 25 years (median 11 years), participated in the study.

### Data collection

Semi-structured interviews were conducted based on an interview guide (Table 1). All participants chose to conduct the interview by telephone. The first author performed all interviews in September 2021. Data were approached inductively and were based on district nurses’ stories about their experiences. To encourage participants to speak freely, follow-up questions were asked such as: ‘Can you explain in more detail?’ and ‘What happened next?’ (Polit and Beck, 2021). Two pilot interviews were conducted, and these were considered to be of good quality and were included in the study. The interviews were recorded using the TapeACall application (TelTech, 2021). All interviews were transcribed verbatim with pauses and words in close connection with the interview (Graneheim and Lundman, 2004). The interviews took between 12 and 35 min to complete.

**Table 1.** Interview guide

What are your experiences of meeting mentally ill young adults in primary health care? Is your experience of a physical meeting different to that of a telephone meeting and, if so, in what way?
What is your experience of the opportunities afforded you to promote the health of mentally ill young adults?
What is your experience of your own competence and knowledge about providing nursing for mentally ill young adults?
What support and resources does your workplace currently provide you with for meeting mentally ill young adults? What support/resources would you like your workplace/employer to provide you with? How often do you come into contact with mentally ill young adults?

### Data analysis

Qualitative content analysis with an inductive approach was used according to Graneheim & Lundman (2004) and Lundman & Hällgren-Graneheim (2017). Both the manifest (the more obvious content of the text) and latent content of a text means that an interpretation is made by the author, but to varying degrees regarding depth and degree of abstraction. The analysis focused on manifest content as much as possible, but certain elements of latent meaning may appear in the categories. In the present study, no overarching themes emerged (Lundman and Hällgren-Graneheim, 2017; Lindgren, Lundman and Graneheim, 2020). The researcher’s pre-understanding and approach were included as part of the process (Polit and Beck, 2021).

The transcripts were read several times to increase the understanding of the content. Subsequently, meaning units were selected that were considered to correspond to the study’s aim. These were then condensed to preserve the core of the content. The condensed meaning units received a code that briefly described the content of the meaning unit where units with the same content received the same code. The codes were grouped into subcategories that then formed categories (Graneheim and Lundman, 2004). Examples of the data analysis are shown (Table 2), and quotes from participants are used in the results to further reinforce the meaning.

**Table 2.** Example of the analysis

Meaning-bearing units	Condensed meaning	Code	Subcategory	Category
‘In my opinion, my opportunities are very limited; these are very short meetings, mainly over the telephone. You might have 12 others queuing so you don’t have the opportunity to speak for half an hour as the individual might sometimes wish’. (6)	Frustration over lack of time preventing one getting to the questions one really wants to ask.	Frustration over lack of time.	The importance of having time for meetings.	A feeling of inadequacy.

## Results

**Figure 1.**
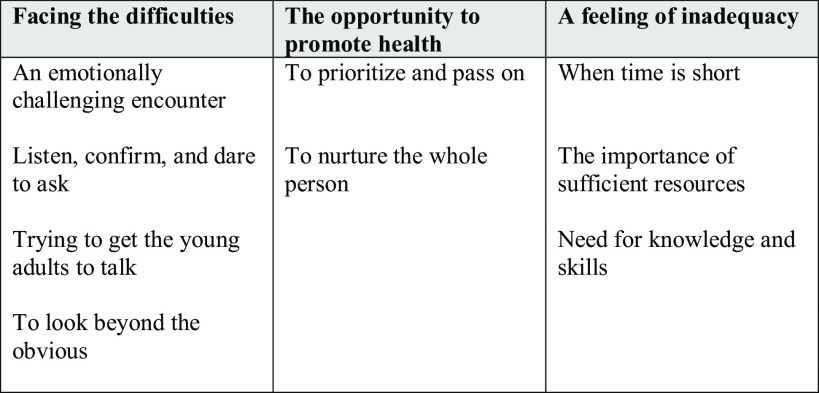
Categories with associated subcategories

The analysis resulted in three categories: *Facing the difficulties*, *The opportunity to promote health*, and *A feeling of inadequacy*. The results are presented in categories with associated subcategories (Figure 1).

### Facing the difficulties

#### An emotionally challenging encounter

The district nurses experienced the meetings with the young adults as emotionally charged. Several district nurses expressed grief and pain at seeing young people suffering from mental illness. The encounters were different from when young adults with physical symptoms sought help, and one district nurse expressed a sense of frustration and helplessness in working with mental illness.

*‘One could probably work with this for an eternity… I don’t know, it seems like we’re just treading water when it comes to this issue’ (4)*

The fact that young adults are faced with many demands and pressures from school and society at large could create a feeling of frustration among the district nurses, which led to the meetings becoming more difficult to handle. The district nurses expressed increased demands and pressures on young people were considered as strong contributing factors to mental illness, and it was felt that today’s society contributes to increased stress.

#### Listen, confirm, and dare to ask

Several of the district nurses reported that young people more often have difficulty asking for help when it comes to mental illness and thus felt that it was important to capture the young adults once they contacted the health centre. The bond with the young adults was often perceived as fragile. Listening and trying to build trust were considered important tools in the conversation. Mapping the family situation and social networks was also an important part of the conversation.

The feeling that it was difficult to decide how best to help the young adults arose in some of the district nurses, which could create frustration. The district nurses emphasized the importance of daring to ask about mental illness, to open up to the conversation and after that create an image of the situation. By routinely asking about factors such as sleep habits, stress, exposure to violence, smoking, alcohol, and substance abuse problems, more people with mental illness could be caught.

*‘To dare to ask questions, to reflect on what that person says. Ask about sleep habits, stress, if you use some forms of substances, and how you think and then get down to the core where you ask about, that you rule out a suicide risk’ (5)*

#### Trying to get the young adults to talk

A factor that influenced the conversation and the relationship between the district nurse and the young adults was how open and contact-seeking they were. If the young adults had difficulty telling how they felt, this could lead to a feeling of frustration among the district nurses as a failure to establish contact and create trust.

#### To look beyond the obvious

The district nurses experienced that mental illness in young adults often was hidden behind other reasons for contacting primary care. Several of the district nurses had experienced that the focus in primary care had previously been on physical needs. The informants experienced that this could lead to the young adults with mental illness in particular sometimes being difficult to identify and a feeling that there were not that many of them. This was about being able to look beyond what they literally said and instead trying to understand what was hidden behind it.

*‘It’s a good conversation where a person state that it is mental illness they suffer from because then they are usually open, seeking help and contact as well as wanting to talk about this while some are not at all… then maybe it creeps out that they actually feel damn bad, that they may even have had those thoughts that they do not want to live anymore’.* (2)

The real reason why the young adults sought help was perceived as masked, and the district nurses experienced that in recent years young adults seemed to have it easier in making contact and directly expressing that they feel mentally ill. The district nurses expressed that the young adult could have been booked for a completely different reason, such as a blood pressure check or for difficulty sleeping, but there was actually mental illness behind the symptoms. It could also be that the young adults mentioned mental illness in passing at the end of a conversation or in connection with a doctor’s appointment that was booked for another reason.

## The opportunity to promote health

### To prioritize and pass on

Assessing, prioritizing, and often guiding the young adults on to other health care providers emerged as a central part of the district nurse’s work with mental illness. The most important task for the district nurses was to make an assessment and then a correct prioritization. Being able to determine what level of care the young adult needed was considered an important job of the district nurse.

*‘We don’t have a flowchart… you just have to try to assess how urgent it is: are they having suicidal thoughts and so on. So, that’s the primary concern. If the situation is so serious that they are having such thoughts, then one shouldn’t wait for an appointment with a primary healthcare provider; at that point, one needs to know where to refer them and so on’.* (9)

The district nurses felt that they had different paths to take when it came to guiding the young adults. The most common was that they sent a referral to mental health for contact with a counsellor or psychologist. How badly the young adult felt determined the extent to which the district nurses were engaged, and the willingness to offer help as soon as possible was present in all participants. Those who expressed thoughts of harming themselves demanded more commitment for the moment than those who sought help for, for example, sleep problems.

*‘My main thought is that, if they don’t have any connections or contacts, the most important thing is that you can offer something concrete, give them a number they can call right away and then I always follow up via their journal’.* (6)

The district nurses expressed that knowing where the young adults could go to get the right help was considered central. Several of the district nurses wanted a more standardized tool to ensure this.

### To nurture the whole person

Promoting health and preventing mental illness was reported to be an important part of the district nurse’s role in primary care.

*‘I think this is a pressing issue, because young adults are our future so of course, it’s important to promote health, which is our most important role in primary healthcare… and, we have much to gain if we can help them with their mental health’.* (5)

The district nurse was considered to have an important function given that they often meet people early on when it comes to mental illness when they are looking for other ailments. Preventing stress in society was raised as an important aspect for preventing mental illness, which involves more actors such as employers.

*‘I’m a great believer in prevention. Preventing stress, preventing people from feeling unwell, catching people at an early stage… prevention will probably demand a great deal of us in future’.* (8)

The district nurses perceived their opportunities to prevent mental illness as limited. If the young adults made contact when they felt very bad, it became a different focus. A difficulty that emerged in several of the interviews was that the district nurses did not meet young people on their own planned visits but only by phone for an initial assessment. Their health-promoting function came only after receiving help from other professional categories such as doctors or psychologists.

Being able to offer quick help, preferably on the same day if deemed necessary, was considered important for the district nurse’s feeling of promoting health. Being able to offer continuity was prioritized but could not always be fulfilled.

The feeling that the work for a district nurse in primary care is categorized and divided appeared among some of the district nurses, and a desire to have a holistic perspective was highlighted. All health centres had access to a sick leave coordinator, and for the district nurses this meant that the young adults with mental illness were referred there if they were on sick leave for a longer period, which meant that the district nurses did not get the chance to promote health.

*‘While as a district nurse one naturally wants to adopt a holistic perspective on the many different pieces, it sometimes feels as if we are simply categorising things. Sometimes I feel that, for a district nurse, there is a certain allure to having this variation’.* (2)

The importance of good collaboration between different professions was emphasized by all district nurses. Other actors such as employers, the municipality, and contributions from society were highlighted by the district nurses as significant. Knowing where to turn and having a safe workplace and people to talk to seemed to strengthen the district nurses and reduce their feelings of helplessness. Cooperation and flexibility among colleagues were key factors in promoting health for the district nurses.

*‘Of course, it demands an organisation that… well, above all here I would say that it is colleagues who have backed up such decisions, creating the opportunity; even if it wasn’t available, we have been quick to alter things to somehow make it happen’.* (3)

The district nurses also often experienced that the young adults did not know where to turn when they were mentally ill. In this way, young adults with mental illness could be captured in a better way. The district nurses emphasized the importance of raising mental illness more at the societal level so that the young adults could dare to express what they feel without being ashamed or embarrassed.

## A feeling of inadequacy

### When time is short

Meeting a young adult with mental illness was considered to require a longer period for the conversation. In the telephone counselling, the district nurses were required to handle a certain number of calls every hour. This could lead to stress and a feeling of not being enough. The district nurses experienced a lack of time as a complicating and limiting factor in their work. It also mattered what day of the week and time of day the young adults called into the health centre. At the beginning of the week, there was less space to give the call the time needed because there were often many people calling.

*‘In my opinion, my opportunities are very limited; these are very short meetings, mainly over the telephone. You might have 12 others queuing so you don’t have the opportunity to speak for half an hour as the individual might sometimes wish’*. *(6)*

### The significance of the nature of the encounter

The experience of the encounter with the young adults by phone or face to face was experienced differently by the district nurses. At a physical meeting, it was easier to read body language and interpret facial expressions, which meant that a different picture of the whole situation emerged. The meeting also had to take longer when it took place physically because the time pressure in telephone counselling is different.

*‘It is somewhat easier to see the patient in front of you; you can assess how they feel in a slightly different way, you can see facial expressions and, in my opinion, whether or not you have eye contact makes an enormous difference’. (8)*

Meeting the young adults through telephone counselling was perceived by several district nurses as an advantage because the young adults could open up differently. As the conversation became more personal and relaxed, it could be easier for the young adults to ask for help. The feeling that the conversation is more disarming over the telephone and the possibility of being more anonymous was highlighted. Some young adults called in felt out of the situation before giving out their social security number.

### The importance of sufficient resources

Having sufficient resources in the workplace was a prerequisite for the district nurses to have opportunity to take care of young adults in a good way. Good access to counsellors and psychologists in the workplace meant that the district nurses felt more secure in their decisions. Lack of resources could lead to district nurses feeling limited and helpless.

*‘… it’s very limited and one can feel slightly helpless, unable to offer the help that this person might need, especially when they might need to talk then and there…’. (6)*

When no appointments for a doctor or mental health professional were available, some of the district nurses booked the young adults to themselves to create a safe zone and then made a new assessment. The district nurses experienced an inequality in resources was considered to exist among the health centres, which in turn leads to unequal care. There was no clear difference in insufficient resources regarding whether the health centre was in an urban or rural area. Sufficient health care professionals at the health centre were linked to greater opportunities to help the young adults.

### Need for knowledge and skills

The district nurses described the need for continuous training and development to be a key factor for the possibility of performing patient-safe work, and all emphasized the importance of knowledge and competence as a decisive factor for meeting with young adults. Many district nurses experienced their knowledge and competence about mental illness as good among those with both longer and shorter experience. The knowledge of how to ask questions to identify mental illness is crucial to how the meeting will go, and the district nurses who expressed a lack of their knowledge and skills more often experienced the meetings as difficult. More knowledge about how to meet a young adult with mental illness through telephone counselling was requested to facilitate the district nurse’s work.

*‘The thing I miss most of all is being able to broaden my knowledge and methods for dealing with these conversations, methods for identifying these young adults when they call us’. (5)*

Several district nurses did not consider that there was a given time for handling the young adults. They wanted clearer routines and flow schedules to achieve more equal care. The district nurses considered that with more education and knowledge, they could contribute more to the care of the young adults, especially at an early stage. More knowledge on how to prevent mental illness was requested.

Having enough knowledge was also considered crucial for the district nurses to reducing the risk of missing someone who has thoughts of harming themselves or someone else. The fact that the manager prioritized the district nurses’ needs for knowledge and further education was considered a prerequisite for being able to run a health centre with a focus on both physical and mental health. However, several district nurses expressed the feeling that their skills were often not used properly.

*‘It seems to me that this is where we really need the organisation with us, in both understanding our training needs and that we are the resource they need to utilise on these issues’. (3)*

## Discussion

Our results showed that the district nurses experienced different challenges and difficulties in meeting the young adults. The lack of time, resources, and knowledge was consistently highlighted by the district nurses as barriers to creating a trusting relationship with the young adults and could lead to a feeling of inadequacy. The importance of listening, confirming, and working in a person-centred manner was emphasized as important for the district nurses’ opportunity to create a caring meeting. This study identified district nurses’ experiences of grief and pain over seeing young adults feeling mentally ill. The meetings were often emotional and hard to deal with because of existential questions about life and death. Lundvall *et al.,* 2019 mention that existential questions can arise in health care professionals during difficult conversation, and it can sometimes be difficult to realize one’s own limitations

The ability to listen and to create trust was perceived by all district nurses in the study as decisive for how the meeting with the young adults was experienced. Creating a relationship with the young adults by confirming and seeing everyone was important. The district nurses experiences are confirmed in studies where the importance of establishing trust and confidence in the relationship by listening, giving time, and being present was emphasized (Björkman *et al.*, 2018; Lundvall *et al.*, 2019). The clinical perspective disappears in the meeting during telephone counselling. When triaging young adults who seek mental health counselling via telephone, this can mean additional obstacles as the clinical view can be decisive (Kaminsky *et al.,* 2017).

The courage to ask questions to identify mental illness in the young adults and to open up for conversation was emphasized by the district nurses. This is also addressed in Björkman *et al.* (2018), where follow-up questions were often asked when the young adults had difficulty talking about their feelings. In the salutogenic approach, listening, confirming, and seeing the whole person in the meeting can increase the district nurses’ sense of meaning and give the patient the same feeling. Being in a context that is understandable, manageable, and meaningful leads to an experience of increased health (Antonovsky, 1987).

The results showed that the district nurses could feel frustrated when they lacked knowledge about how best to help the young adults with mental illness who made contact with them. Some district nurses felt that the only thing they had to offer was the ability to listen. This is confirmed by Obando-Medina *et al.*, (2014) where feelings of powerlessness among health care professionals were reported. They could feel frustrated, incompetent, and depressed when they could not solve the young adults’ problems. However, the district nurses expressed a sense of relief when they managed to reach out and calm the young adults by listening. The results described the experience of uncertainty about their own efforts in the meeting, and there was a fear of missing something serious such as suicidal thoughts. The same experience emerged in Lundvall *et al.*, (2019), where feelings of insecurity were reported. To deal with these feelings, the district nurse could offer renewed contact soon after the first meeting.

When the young adults had difficulty expressing their feelings, this could lead to frustration among the district nurses because it was perceived as a failure to create a feeling of trust. Lundvall *et al.* (2019) confirm that conversing with young adults means searching for their innermost thoughts and feelings much like a detective. In Janlöv *et al.* (2018), the district nurses looked for both verbal and non-verbal signs of mental illness in the meeting, which was perceived as particularly important when the patients were emotionally careful and hesitant. Difficulties could be associated with a feeling of shame and guilt due to the stigma surrounding mental illness (Björkman *et al.*, 2018). The results of the present study emphasize the importance of young adults knowing where to turn. Promoting awareness and knowledge about mental health in young adults was considered an important part of district nurses work to reduce stigma.

District nurses often experienced that the mental illness was masked behind other ailments of a more physical nature, such as difficulty sleeping or high blood pressure. This placed higher demands on the district nurses’ ability to identify the young adults. Several studies confirm this experience that patients come to the health centre booked for a physical ailment but that it later emerges during the visit that the real cause was mental illness (Obando-Medina *et al.*, 2014; Björkman *et al.*, 2018; Janlöv *et al.*, 2018; Lundvall *et al.*, 2019). Björkman and Salzmann-Erikson (2018) believe that some patients who applied for mental illness were ashamed to talk about their problems, which was shown by the fact that they first did not talk about the real reason why they called. According to Obando-Medina *et al.* (2014), before an attempted suicide, young adults often seek primary care for physical symptoms such as headache or back pain. Lundvall *et al.* (2019) found that health care professionals often experience that the young adults mask their real feelings.

The results also described the district nurses’ sense of benefits for young adults being able to contact primary care via digital means. Bradford and Rickwood (2014) believe that digital tools can help young adults with mental illness because they can talk about their problems, especially if they are perceived as embarrassing. However, there is a link between mental illness among social media and depression among young adults. Social media can increase the risk of young adults suffering from mental illness and lead to low self-esteem, depressive symptoms, and suicide (Radovic *et al.,* 2017; O´Reilly *et al.,* 2018).

The district nurses experienced their opportunities to promote health as limited in the first meeting with the young adults. Their health-promoting function came mainly after contact with a psychologist or doctor. Rönngren *et al.* (2018) argue that district nurses can make a difference for young adults with mental illness by providing knowledge about lifestyle factors. The district nurses emphasized that they were an asset in the work with mental illness that was sometimes not taken advantage of. Those who worked in the lifestyle clinic at the health centre experienced greater opportunities to be able to make a difference. Janlöv *et al.* (2018) describes how mental illness among young adults is often related to lifestyle issues such as stress in working life or various forms of addiction. Gall *et al.* (2016) demonstrated that a healthy lifestyle can promote mental health.

It was important for the district nurses that the young adults received help and care at the health centre whenever possible because it was felt to be safer for the young adults when they did not have to leave the health centre, especially if the health centre was in the countryside. In Obando-Medina *et al.* (2014), the young adults who were referred to another care unit did not always go there. This could be because a relationship was already established with the health care professionals at the health centre, which meant that the patients did not want to change health care providers. Turning to primary care instead of a psychiatric clinic could be perceived by patients as less stigmatizing.

The district nurses in this study called for better collaboration between primary care, outpatient care, and inpatient care. It could be perceived as difficult to get in touch and a feeling that referrals to psychiatry were often rejected. Difficulties in obtaining help from specialized psychiatry have been highlighted previously (Bagayogo *et al.*, 2018; Janlöv *et al.*, 2018). This can lead to patients being sent back and forth with no one taking responsibility (Björkman *et al.*, 2018).

Our study showed a feeling of inadequacy among the district nurses in the encounter with the young adults with mental illness. Lack of time, knowledge, and resources were an obstacle to being able to offer the right care. This could lead to feelings of helplessness, frustration, and insecurity. Obando-Medina *et al.* (2014) highlighted time and knowledge as crucial for the ability to see beyond the physical symptoms. Deficiencies in time and knowledge could lead to health care professionals ignoring signs of mental illness, and this makes it difficult to integrate mental health into primary care (Bagayogo *et al.*, 2018).

The district nurses emphasized the importance of sufficient knowledge to be able to meet the young adults with mental illness. When knowledge was lacking, this could lead to a feeling of uncertainty about their assessments. This is confirmed in several studies where knowledge regarding mental illness is often perceived as deficient by health care professionals in primary care, and more knowledge is considered necessary to be able to offer safe and secure care (Leahy *et al.*, 2013; Obando-Medina *et al.*, 2014; Björkman *et al.*, 2018 and Janlöv *et al.*, 2018).

### Strengths and limitations

The advantage of a qualitative study is the opportunity to go in depth and thus gain knowledge about how the phenomenon being investigated is experienced (Polit and Beck, 2021). Strategic selection was implemented in this study to strengthen credibility. There was a wide range in age and work experience, which is an advantage for the breadth of the results. However, there was no distribution in terms of gender as all district nurses were women, which might affect the generalizability (Graneheim and Lundman, 2004). It is the quality of the material collected that will determine this number (Graneheim *et al.*, 2017; Polit and Beck, 2021). Time frame for the interviews varied, anyhow the quality of data material considered was of importance. The fact that health centres from the southeast and northern part of Sweden and from both urban and rural areas were selected strengthen the study’s credibility as a greater variety of experiences was obtained. All health centres were regionally controlled, which could affect the study’s transferability to, for example, private health centres with other conditions (Polit and Beck, 2021).

Semi-structured individual interviews based on an interview guide were conducted. The amount of data needed to answer the purpose of the study in a credible way depends on the complexity of what is being studied, but also on the quality of the data collected (Graneheim and Lundman, 2004). All informants conducted the interviews over the phone, which meant that the author could not see the participants during the interviews. The interviews were transcribed in close connection with the interviews to ensure that all nuances of the conversation were maintained (Polit and Beck, 2021).

To support the dependability of the study, data collection was performed during one month (Graneheim and Lundman, 2004). A couple of days before the interviews, the participants received the main questions in the interview guide in order to provide an opportunity for in-depth reflection during the interview. Interviews are an ongoing process that can affect the follow-up questions that are asked, and this can affect the trustworthiness of the study (Graneheim and Lundman, 2004).

To strengthen the credibility of the study, the texts were read repeatedly to ensure that no relevant data were excluded, and that no irrelevant data were included. Credibility was strengthened by presenting representative quotations from the transcribed text in the results, which can strengthen the transferability. According to rigour and trustworthiness in the data analysis, both authors discussed the result until consensus (Graneheim and Lundman, 2004). An inductive approach was applied where patterns, differences, and similarities were in focus, which led the author to try to avoid general summaries and superficial descriptions (Graneheim, Lindgren and Lundman, 2017). An example of the analysis process is shown (Table 2), which can be seen as a kind of reflection that strengthens trustworthiness (Graneheim, Lindgren and Lundman, 2017).

The author’s experience from work in primary care is that more and more young adults seek care for mental illness. The fact that the author and the district nurse together created the interview means that the questions asked were affected by the author’s pre-understanding, which in turn affected the study’s credibility positively (Graneheim, Lindgren and Lundman, 2017).

## Conclusion

Lack of knowledge, resources, and time hindered the district nurses’ health promotion work in meeting with young adults with mental illness, which created experiences of stress, frustration, and inadequacy. Building a trusting relationship and having the prerequisites for building that trust were crucial. If given the tools needed, the district nurses perceive they could manage the important role being a resource and take a greater responsibility to guide young adults on the right path. Continuous training and the opportunity to provide sufficient time were emphasized as important for the district nurse’s ability to make correct initial assessments and provide good person-centred care.

## Clinical implications

The results of this study fill a knowledge gap because few studies have been conducted with a focus on district nurses’ experiences of young adults with mental illness in primary care. The results could be used to show managers in primary care how district nurses experience these meetings and how they need the right conditions for a good meeting where they can feel confident in their decisions and gain a sense of adequacy. Experiences of insecurity, frustration, and helplessness among the district nurses might then decrease.

## References

[ref1] Antonovsky A (1987) Unraveling the mystery of health. How people manage stress and stay well. San Francisco: Jossey-Bass.

[ref2] Bagayogo IP , Turcios-Wiswe K , Taku K , Peccoralo L and Katz CL (2018) Providing mental health services in the primary care setting: the experiences and perceptions of general practitioners at a New York city clinic. Psychiatric Quarterly 89, 897–908. 10.1007/s11126-018-9587-2 29968148

[ref3] Björkman A , Andersson K , Bergström J and Salzmann-Erikson M (2018) Increased mental illness and the challenges this brings for district nurses in primary care settings. Issues in Mental Health Nursing 39, 1023–1030. doi: 10.1080/01612840.2018.1522399. 30624130

[ref4] Björkman A and Salzmann-Erikson M (2018) When all other doors are closed: Telenurses´experiences of encountering care seekers with mental illnesses. International Journal of Mental Health Nursing 27, 1392–1400. 10.1111/inm.12438 29383820

[ref5] Bradford S and Rickwood D (2014) Young people’s views on electronic mental health assessment: prefer to type than talk? Journal of Child and Family Studies 24, 1213–1221. 10.1007/s10826-014-9929-0 PMC4412384 25960628

[ref6] Gall SL , Sanderson K , Smith KJ , Patton G , Dwyer T and Venn A (2016) Bi-directional associations between healthy lifestyles and mood disorders in young adults: the Childhood Determinants of Adult Health Study. Psychological Medicine 46, 2535–2548. 10.1017/S0033291716000738 27338017

[ref7] Graneheim UH and Lundman B (2004) Qualitative content analysis in nursing research: concepts, procedures and measures to achieve trustworthiness. Nurse Education Today 24, 105–112. 10.1016/j.nedt.2003.10.001 14769454

[ref8] Graneheim UH , Lindgren B and Lundman B (2017) Methodological challenges in qualitative content analysis: a discussion paper. Nurse Education Today 56, 29–34. 10.1016/j.nedt.2017.06.002 28651100

[ref9] Haddad M , Walter P and Tylee A (2007) District nursing staff and depression: a psychometric evaluation of Depression Attitude Questionnaire findings. International Journal of Nursing Studies 44, 447–56. 10.1016/j.ijnurstu.2006.07.005 16979641

[ref10] Janlöv AC , Johansson L and Clausson EK (2018) Mental ill-health among adult patients at healthcare centres in Sweden: district nurses experiences. Scandinavian Journal of Caring Sciences 32, 987–996. 10.1111/scs.12540 29131370

[ref11] Kaminsky E , Röing M , Björkman A and Holmström IK (2017) Telephone nursing in Sweden: a narrative literature review. Nursing & Health Sciences 19, 278–286. 10.1111/nhs.12349 28618087

[ref12] Leahy D , Schaffalitzky E , Armstrong C , Bury G , Cussen-Murphy P , Davis R , Dooley B , Gavin B , Keane R , Keenan E , Latham L , Meagher D , McGorry P , McNicholas F , O’Connor R , O’Dea E , O’Keane V , O’Toole T , Reilly E and Ryan P (2013) Primary care and youth mental health in Ireland: qualitative study in deprived urban areas. BMC Family Practice 14. DOI: 10.1186/1471-2296-14-194. PMC3880165 24341616

[ref13] Lindgren BM , Lundman B and Graneheim UH (2020) Abstraction and interpretation during the qualitative content analysis process. International Journal of Nursing Studies 108, 103632. doi: 10.1016/j.ijnurstu.2020.103632. 32505813

[ref14] Liu NH , Daumit GL , Dua T , Aquila R , Charlson F , Cuijpers P , Druss B , Dudek K , Freeman M , Fujii C , Gaebel W , Hegerl U , Levav I , Munk Laursen T , Ma H , Maj M , Elena Medina-Mora M , Nordentoft M , Prabhakaran D , Pratt K , Prince M , Rangaswamy T , Shiers D , Susser E , Thornicroft G , Wahlbeck K , Fekadu Wassie A , Whiteford H and Saxena S (2017) Excess mortality in persons with severe mental disorders: a multilevel intervention framework and priorities for clinical practice, policy and research agendas. World Psychiatry 16, 30–40. 10.1002/wps.20384 28127922 PMC5269481

[ref15] Lundman B and Hällgren Graneheim U (2017) Kvalitativ innehållsanalys. In Granskär M and Höglund-Nielsen B , Editors, Tillämpad kvalitativ forskning inom hälso- och sjukvård. Studentlitteratur, pp. 219–234. Sweden.

[ref16] Lundvall M , Lindberg E , Hörberg U , Palmér L and Carlsson G (2019) Healthcare professionals’ lived experiences of conversations with young adults expressing existential concerns. Scandinavian Journal of Caring Sciences 33, 136–143. 10.1111/scs.12612 30152541

[ref17] Ministry of Social Affairs and Sweden´s municipalities and regions. (2021) *Good and close care. A transformation of health care with primary care as a hub.* Retrieved 7 February 2022 at: https://skr.se/download/18.71a6757217b07d9b39fb939b/1629783112706/God_och_nara_vard_2021_uppdaterad.pdf

[ref18] National Board of Health and Welfare. (2021) *National guidelines for care for depression and anxiety disorders. Support for governance and management.* Retrieved 7 February 2022 at: https://www.socialstyrelsen.se/globalassets/sharepoint-dokument/artikelkatalog/nationella-riktlinjer/2021-4-7339.pdf

[ref19] O’Reilly M , Dogra N , Whiteman N , Hughes J , Eruyar S and Reilly P (2018) Is social media bad for mental health and wellbeing? Exploring the perspectives of adolescents. Clinical Child Psychology and Psychiatry 23, 601–613. 10.1177/1359104518775154 29781314

[ref20] Obando-Medina C , Kullgren G and Dahlblom K (2014) A qualitative study on primary health care professionals’ perceptions of mental health, suicidal problems and help-seeking among young people in Nicaragua. BMC Family Practice 15, 129. 10.1186/1471-2296-15-129 24989871 PMC4112650

[ref21] Olasoji M , Maude P and Cross W (2020) Experiences of mental health nurses working in general practice: a qualitative study. Contemporary Nurse 56, 266–279. doi: 10.1080/10376178.2020.1841013. Epub 2020 Nov 19. PMID: 33086987. 33086987

[ref22] Polit DF and Beck CT (2021) Nursing research: Generating and assessing evidence for nursing practice. Philadelphia, PA: Wolters Kluwer.

[ref23] Radovic A , Gmelin T , Bradley DS and Miller E (2017) Depressed adolescents positive and negative use of social media. Journal of Adolescence 55, 5–15. 10.1016/j.adolescence.2016.12.002 27997851 PMC5485251

[ref24] Rönngren Y , Björk A , Kristiansen L , Haage D , Enmarker I and Adudulv Å (2018) Meeting the needs? Perceived support of a nurse-led lifestyle programme for young adults with mental illness in a primary health-care setting. International Journal of Mental Health Nursing 14, 390–399. 10.1111/inm.1233 28374967

[ref26] Swedish Nurses’ Association. (2019) Competency description advanced level district nurse. Retrieved 7 February 2022 at: https://www.swenurse.se/download/18.9f73344170c0030623175b/1584023673165/kompetensbeskrivning%20distriktssk%C3%B6terska%202019.pdf

[ref27] TelTech. (2021) TapeACall. Retrieved 16 September 2021 at: https://www.tapeacall.com

[ref28] Tong A , Sainsbury P and Craig J (2007) Consolidated criteria for reporting qualitative research (COREQ): a 32-item checklist for interviews and focus groups. Journal of the International Society for Quality in Health Care 19, 349–357. 10.1093/intqhc/mzm042 17872937

[ref29] World Health Organization. (2021a) *Mental health.* Retrieved 7 February 2022 at: https://www.who.int/health-topics/mental-health#tab=tab_1

[ref30] World Health Organisation. (2021b) Comprehensive mental health action plan 2013-2030. WHO. Retrieved 8 April, 2023 at: https://www.who.int/publications/i/item/9789240031029

